# Use of regional transmural agreements to support the right care in the right place for patients with chronic heart failure—a qualitative study

**DOI:** 10.1007/s12471-022-01740-5

**Published:** 2022-12-12

**Authors:** Stefan L. Auener, Patrick P. T. Jeurissen, Dirk J. A. Lok, Huug J. van Duijn, Petra E. J. van Pol, Gert P. Westert, Simone A. van Dulmen

**Affiliations:** 1 Radboud University Medical Center, Radboud Institute for Health Sciences, Scientific Center for Quality of Healthcare (IQ Healthcare), Nijmegen, The Netherlands; 2 Department of Cardiology, Deventer Hospital, Deventer, The Netherlands; 3 Knooppunt Ketenzorg, Leiden, The Netherlands; 4 Department of Cardiology, Onze Lieve Vrouwe Gasthuis, Amsterdam, The Netherlands

**Keywords:** Chronic heart failure, Transmural care, Collaboration, Primary healthcare

## Abstract

**Background:**

Chronic heart failure (CHF) poses a major challenge for healthcare systems. As these patients’ needs vary over time in intensity and complexity, the coordination of care between primary and secondary care is critical for them to receive the right care in the right place. To support the continuum of care needed, Dutch regional transmural agreements (RTAs) between healthcare providers have been developed. However, little is known about how the stakeholders have experienced the development and use of these RTAs. The aim of this study was to gain insight into how stakeholders have experienced the development and use of RTAs for CHF and explore which factors affected this.

**Methods:**

We interviewed 25 stakeholders from 9 Dutch regions based on the Measurement Instrument for Determinants of Innovations framework. Interview recordings were transcribed verbatim and analysed through open thematic coding.

**Results:**

In most cases, the RTA development was considered relatively easy. However, the participants noted that sustainable use of the RTAs faced different complexities and influencing factors. These barriers concerned the following themes: education of primary care providers, referral process, patients’ willingness, relationships between healthcare providers, reimbursement by health insurance companies, electronic health record (EHR) systems and outcomes.

**Conclusion:**

Some complexities, such as reimbursement and EHR systems, are likely to benefit from specialised support or a national approach. On a regional level, interregional learning can improve stakeholders’ experiences. Future research should focus on quantitative effects of RTAs on outcomes and potential financing models for projects that aim to transition care from one setting to another.

**Supplementary Information:**

The online version of this article (10.1007/s12471-022-01740-5) contains supplementary material, which is available to authorized users.

## What’s new?

Regional transmural agreements (RTAs) are a way of formalising agreements among different healthcare providers to provide a continuum of care.

- Although the development of RTAs between primary and secondary care providers for chronic heart failure seemed relatively easy, sustainable use of these agreements faced different barriers that needed to be addressed.
- Overarching cooperatives of general practitioners (care groups) or other umbrella organisations can play a pivotal role in supporting the use of RTAs.
- Interregional learning can support healthcare professionals involved in RTA development and use.
- More evidence on the effect of RTAs on both the quality and costs of care is needed.

## Introduction

Chronic heart failure (CHF) poses a major challenge for our healthcare systems because of its morbidity, mortality, increasing prevalence and costs [1, 2]. The needs of patients with CHF often vary over time due to the erratic and unpredictable nature of the disease [3]. In addition, studies have shown that there is severe underdiagnosis among CHF patients [4] and that palliative care for this patient group can be improved upon [3]. Despite the fact that patients’ needs vary, stable patients with CHF on optimal therapy can safely be referred from secondary care (SC) back to primary care (PC) [5, 6]. This requires coordination and collaboration between healthcare providers throughout the entire patient journey.

To support the continuum of care needed to improve care for patients with CHF in the Netherlands, a framework called the National Transmural Agreement (NTA) Heart Failure was developed [7]. This NTA was based on the national guidelines Heart Failure of the Dutch College of General Practitioners published in 2010 (*tweede herziene NHG-Standaard Hartfalen), *the European Society of Cardiology (ESC) Standards for delivering heart failure care (2011) and the ESC guidelines for the diagnosis and treatment of acute and chronic heart failure (2012) [8–10]. The NTA requires multidisciplinary transmural organisation of care for CHF patients, provides guidance for regions to develop regional transmural agreements (RTAs) between healthcare professionals and addresses topics such as the diagnostic process, referral to SC and back to PC and the coordination of palliative care [11]. More information on the NTA Heart Failure is provided in Table S1 in the Electronic Supplementary Material.

It is unknown how stakeholders have experienced working with these RTAs and in which areas they have noticed facilitators and barriers. Therefore, we aimed to evaluate how stakeholders have experienced the development and use of RTAs for CHF.

## Methods

We conducted semi-structured interviews to study the experiences of stakeholders with the development and use of RTAs for CHF and explore any factors that affected this development and use. The study protocol was assessed by The Medical Ethics Committee of the Radboud University Medical Centre determined that this research was not subject to the Dutch Medical Research Involving Human Subjects Act (*Wet medisch-wetenschappelijk onderzoek met mensen)* (registration number 2020-7182). Therefore, ethical approval was not needed. This study complied with the Declaration of Helsinki.

### Participants

Participants were invited through the professional networks of the authors and the Connect Project Group of the Netherlands Society of Cardiology (*Nederlandse Vereniging voor Cardiologie*). Through purposive sampling, 35 participants were invited. Additional participants were recruited through snowball sampling. All participants provided written informed consent prior to the interview. Interviews were conducted by video conference or telephone and lasted 24–65 min. We interviewed 25 stakeholders (11 males and 14 females) from 9 Dutch regions. Two participants made a deliberate decision not to join or start an RTA at the time. Tab. 1 shows the professions of the participants.

**Table 1:** Professions of participants interviewed

Profession	Participants (*n*)^a^
Cardiologist	9
General practitioner	4
Nursing specialist/Nurse	2
Nurse practitioner in primary care	1
Project leader RTA/coordinator care group	7
Healthcare insurer	2
Member of atient association p	1
Netherlands Society of Cardiology Connect Project Group	2

*RTA* regional transmural agreement

^a^ Some participants had multiple functions within their region

### Interviews

The interview guide was based on the Measurement Instrument for Determinants of Innovations (MIDI) framework and was expanded upon in subsequent interviews based on the responses in previous interviews; the topic guide for the interviews is provided in Table S2 in the Electronic Supplementary Material. The MIDI consists of four determinants that are important for successful implementation: the innovation, the adopting person, the organisation and the social-political context [12]. The majority of the interviews were conducted by two researchers (SA, SvD).

### Data analysis

Interviews were recorded, transcribed verbatim and analysed through open thematic coding in ATLAS.ti (ATLAS.ti Scientific Software Development GmbH, Berlin, Germany). The first 9 interviews were coded independently by two investigators (SA, SvD) and checked for discrepancies. In case of discrepancies, coding was discussed until consensus was reached. After the coding system was established, one researcher (SA) coded the remaining interviews.

## Results

Participants involved in the development of the RTAs (*n* = 12) reported very few barriers for the development of RTAs. However, the group of participants had a different view on the use of the RTAs. While some participants experienced very few barriers in using the RTAs, others thought there were several barriers that, despite much effort, could not be overcome. These barriers concerned the following themes: education of PC providers, referral processes, patients’ willingness, relationships between healthcare providers, reimbursement, electronic health record (EHR) systems and outcomes. Tab. 2 contains illustrative quotes from the interviews. The themes are described below (see Table S3 in the Electronic Supplementary Material for an extensive description of each theme).

**Table 2:** Illustrative quotes from interviews grouped per theme

Theme	Illustrative quote
Education of PC providers	“Because it is a fairly complex syndrome … these are quite small numbers in practice, so you don’t get a real routine with it.” “And if that is not enough, I think it is important that they can still get access [to SC consultation] and that this is not immediately a contact, which then leads to referral back to SC, and all the costs that go with it, but just accessible collegial advice.”
Referral from SC to PC	“You only know the really bad and the really good [GPs], but you don’t know the others. And you only know that because you have been getting referrals for years.”
Patients’ willingness	“… patients are very positive about the heart failure clinic. Actually, people who are under the control of a heart failure clinic feel very safe.” “And yes, we also say: ‘If you can’t get any further with the GP or if there is a problem reaching the GP or anything, you can also contact us immediately.’ So, they do have a bit of a safety net in that sense. And with this, a larger part of the patients are happy to do it that way.”
Relationships between healthcare providers	“But they [physicians that started an RTA] also have a following and they repeat that at times: ‘We have to start slowly. Some people have no feeling at all with heart failure, some do. You simply can’t force it.’ When setting up the project, they also asked their following whether they agree that we will continue this. The supporters agreed. And I think that is very essential to take a step”
Reimbursement	“Then you only have to deal with the GPs and the specialists. And the moment the GP says ‘Yes, but then we also want to be reimbursed for that’, then the health insurance company comes in and then the business case comes in and then comes the baseline measurement and measuring end points. And then it becomes extremely complex.” “Now we depend on whether we get it for another 3 years and in the back of your mind you are afraid that the health insurance companies will say: ‘Okay, that is the end of the extra money.’” “Are you reliable? Not only as a person but also as an organisation? Is there not a different wind blowing every year that makes them think: I am going to do something now, but can I keep doing it next year? So, those are extremely important preconditions for these kinds of processes.”
EHR systems	“Yes, the classification and storage of information within GP information systems shocked me. And then I also understood it better: it is not reluctance on the part of a GP; he should be facilitated better.”
Outcomes	“Where there are certainly still opportunities for improvement, and what we are also going to work on, is that we will provide the practices with benchmark information in order to also give them more feedback on ‘How many patients have you included?’ And thus, hopefully, stimulate that the patients are better identified. And that they [PC providers] move more towards that expected prevalence.”

*PC* primary care,* SC* secondary care, *GP* general practitioner, *RTA* regional transmural agreement, *EHR* electronic health record

### Education of primary care providers

The participants frequently emphasised the importance of education of PC providers. This education was considered essential for treatment of CHF patients in PC, and providing it was often the first action of regions after the development of the RTA. The participants often reported that CHF is perceived as complicated to treat and that knowledge and awareness of CHF vary in PC. They also indicated that the number of CHF patients per PC clinic was low. Furthermore, the low prevalence was considered a barrier to gain additional education. Education participation rates of PC providers varied by region. Most participants mentioned the importance of easy consultation options to mitigate knowledge and experience gaps. Besides knowledge of the disease, education was aimed at informing participants on care pathways, consultation abilities, and changes due to the RTA.

### Referral from secondary to primary care

The NTA Heart Failure provides guidance for the development of RTAs, which has resulted in different criteria for referral to PC. Besides these different criteria in the RTAs, trust in the competencies in PC sometimes affected referrals. Several cardiologists noted that they were unaware of which PC clinic had affinity and experience with CHF patients and which did not. In some cases, a lack of trust in PC skills prevented cardiologists to refer patients to PC. However, most cardiologists reported they followed the criteria in the RTA, regardless of the PC clinic.

### Patients’ willingness

Several participants mentioned that patients are sometimes unwilling to be referred back to PC due to high satisfaction with the care at specialised CHF clinics. The participants noted that patient education on care pathways and the ability for patients to contact the CHF clinic could improve patients’ willingness to be referred back to PC.

### Relationships between healthcare providers

The interviewees reported that RTAs must be in line with the skills and facilities, such as communication methods, of both PC and SC providers. Therefore, it was important that the initiators, who developed the RTA, had good relationships with their colleagues. This remained essential after the development of the agreements to ensure continued participation. For example, cardiologists specialised in CHF needed to monitor whether their colleagues with other specialties than CHF also referred patients according to the RTA.

In addition, it was frequently mentioned that ‘knowing each other’ was an important facilitator for the collaborative elements of the RTA. This created a better understanding of each other’s abilities, challenges and level of trust. These personal contacts became scarcer as more healthcare providers were present in the region. Trust between providers and health insurance companies was also considered to be important.

### Reimbursement

Many participants thought arranging reimbursement of additional activities related to the RTA was a major hurdle. These additional activities referred to education, identification of patients and (administrative) support for implementation. While some regions obtained reimbursement for all activities, others did not. Healthcare providers were often unfamiliar with the reimbursement processes and experienced them as complicated. Although the employees of health insurance companies reported that funds for project leaders were available, these were not always accessed. Moreover, health insurance companies’ policies differed on whether and how reimbursements were arranged. A facilitating factor for receiving reimbursement was the presence of a well-organised care group and/or another umbrella organisation supporting administrative processes.

### Electronic health record systems

The ability to share data with professionals in other settings was perceived as a facilitating factor for the implementation of the RTA and the improvement of the quality of care. The participants reported a lack of interoperability or different EHR systems as complicating factors for data sharing. These factors complicated information exchange between healthcare providers and hindered evaluations.

According to the participants, identification of patients with CHF is an important aspect of RTAs. This includes the identification of stable patients in SC who may be referred to PC and undiagnosed patients in PC. The participants noted that time-consuming manual review of patient records was necessary to find a limited number of patients. In some cases, this identification process was supported by a care group or software solution.

### Outcomes

Most regions evaluated the effects of the RTA. However, the outcome measure selections were heterogenous. The number of referrals to PC was often evaluated as part of reimbursement agreements and showed different results. While some participants noted that initial targets had been achieved, others reported that initial expectations were too high. Other outcome measures were process indicators, such as the registration of physiological parameters. Evaluations were used to identify areas for improvement. Several participants mentioned that data on healthcare provider level could be used to guide and motivate PC clinics by providing benchmark information. Although regions intended to evaluate patient experience, little information was available.

## Discussion

In general, stakeholders experienced very few barriers for the development of RTAs. However, the participants noted that actual utilisation of RTAs came with a variety of complexities. These complexities were often multidisciplinary in nature and required substantial organisational support and cooperation in order to proceed with further implementation. Although these complexities were common in many regions, the degree to which these were experienced as problematic differed.

Many of the issues identified are not exclusive to the use of RTAs but are also common in comparable projects [13–15]. A shift from SC to PC requires extensive collaboration that is not self-evident. Berendsen et al. concluded that lack of time and financial compensation, complexity and little support from colleagues are important barriers for a collaboration between PC and SC [13]. These barriers were also reported in the present study.

Smeets et al. showed that a lack of awareness and experience may hinder the treatment of CHF patients in PC [14]. We found that the lack of awareness and experience was mainly attributable to the low number of CHF patients per PC clinic. This stressed the need for easy options to consult SC providers. Such a feature, whether through telephone or the EHR system, was appreciated by participants and was facilitated in several regions. This finding does not seem to be limited to cardiologists but also to other medical specialties [15].

Reimbursement is often described as an important barrier for projects in which hospital care is substituted by PC [13, 16]. In our study, participants from most regions described the process of arranging reimbursement as cumbersome. The low number of patients per PC clinic sometimes resulted in negative business cases, in which savings did not outweigh the investments needed. Furthermore, several health insurance companies had different policies and visions on whether and how projects such as the development of RTAs should be reimbursed. Since many healthcare providers are dependent on multiple health insurance companies, this makes the process of arranging reimbursement more complex. Sometimes, there were concerns about the continuation of reimbursement after the initial reimbursement. Although this was not always the case, it is exemplary of the low trust levels between health insurance companies and healthcare providers in the Netherlands [13, 17]. Multi-year contracts and lump-sum payment models may support transmural projects but have their own limitations [18].

While the increasing prevalence of stable CHF may tilt the business case in the right direction, it is still uncertain whether a larger number of CHF patients per PC clinic will consistently result in monetary savings. However, a key component of the RTA, which is education, may not only result in monetary gains but also in health gains, since more awareness and knowledge among PC providers can decrease underdiagnosis and improve patient-centred care. Especially the latter is difficult to measure and price accordingly [19].

### Implications

Our study showed that many issues described in the implementation literature are still current. However, it also demonstrated that many healthcare providers are unaware of these issues and need support. A lack of support may result in decreased devotion, but devotion is essential for increasing collaborative care [13]. While certain activities such as education and the process of arranging reimbursement were organised by a few enthusiastic healthcare providers in some regions, other regions had well-organised organisations that supported these activities and processes. Representatives of the latter regions often reported less problems. Initiators of RTAs should be aware of the complexities and ensure that proper support for them is in place.

Although the participants acknowledged the complexities of the issues, several of them reported they felt as if they were reinventing the wheel and they were curious how other regions dealt with certain barriers. This suggested that exchanging experiences and solutions between regions may support implementation of RTAs and allow for interregional learning. This approach is likely to be helpful for issues that exist on a regional level, such as relationships between healthcare providers and education of PC providers. Other issues, such as reimbursement and workload of PC providers, are more general and require broader discussions on a national level. In addition, more evidence on the effect of RTAs on the quality and costs of healthcare may result in more robust business cases, which allows for easier reimbursements and increased motivation of providers to initiate and participate in RTAs.

### Strengths and limitations

A major strength of this study is the variety of professions of the interviewees from different regions. Previous studies on collaborative care or substitution of care have often highlighted one perspective [13, 14, 20]. An integral view of these perspectives further demonstrates the complexity of barriers that arise when policy is aimed at facilitating the right care in the right place. In addition, our results are likely to have a high degree of transferability to other RTAs and collaborative projects.

Due to the COVID-19 crisis and subsequent increased workload, the number of healthcare providers available for interviews was limited. This resulted in a limited number of participating PC providers. However, we interviewed a total of 4 project leaders and coordinators who were employed by PC organisations and were involved in the use of RTAs.

## Conclusion

On paper, the development of an RTA between healthcare providers seems feasible. However, there were many factors that affected the actual use of the RTAs for CHF and the commitment of stakeholders, which resulted in complexities that were not easily addressed. Interregional learning and discussions on a national level may support further development and use of RTAs.

## Supplementary Information

**Table S1** Background on National Transmural Agreement Heart Failure and regional transmural agreements

**Table S2** Topic guide for interviews

**Table S3** Extensive description of themes
