# Supplementary material for: Use of regional transmural agreements to support the right care in the right place for patients with chronic heart failure—a qualitative study
Source: Neth Heart J. 2022 Dec 12;31(3):109–16. doi: 10.1007/s12471-022-01740-5 (PMC9742644; doi:10.1007/s12471-022-01740-5)
Supplement: Supplementary file 1 — Table S1 Background on National Transmural Agreement Heart Failure and regional transmural agreements [file 12471_2022_1740_MOESM1_ESM.docx]

**Table S1** Background on National Transmural Agreement Heart Failure and regional transmural agreements

| The needs of patient with chronic heart failure differs among patients and across time due to the erratic trajectory of many patients and the high degree of multimorbidity. During some periods, treatments and check-up visits in secondary or tertiary care are indicated. However, patients on optimal therapy may be referred back to primary care. To improve the entire patient journey while simultaneously stimulating the right care at the right place, the Dutch Society for Cardiology (in Dutch: Nederlandse Vereniging voor Cardiologie), the Dutch College of General Practitioners (in Dutch: Nederlands Huisartsen Genootschap), the umbrella organisation of health insurers (in Dutch: Zorgverzekeraars Nederland), and the patient association for cardiovascular diseases (in Dutch: Harteraad) developed the “National Transmural Agreement (NTA): heart failure” in 2015. The NTA is based on national guidelines, the guidelines of the European Society of Cardiology, and the Standards for delivering heart failure care from the European Society of Cardiology. The NTA prescribes the multidisciplinary transmural organisation of care for patients with chronic heart failure and provides guidance for regions to develop Regional Transmural Agreements (RTAs) between healthcare professionals. This regional approach allows for priority setting and agreements based on the needs and circumstances of the region. While these RTAs are not obligatory, there has been a consistent increase in the number of regions that developed an RTA. Currently, there are 20 regions that developed and published an RTA for chronic heart failure.  The following themes are addressed by the National Transmural Agreements:   - Healthcare professionals involved and their responsibilities - Additional diagnostics and treatment advice - Referral back to primary care - Education and lifestyle advice - Heart failure rehabilitation - Check-up visits - Role of the practice nurse in primary care - Role of the heart failure nurse - Registration - Palliative care |
| --- |
