# Supplementary material for: Use of regional transmural agreements to support the right care in the right place for patients with chronic heart failure—a qualitative study
Source: Neth Heart J. 2022 Dec 12;31(3):109–16. doi: 10.1007/s12471-022-01740-5 (PMC9742644; doi:10.1007/s12471-022-01740-5)
Supplement: Supplementary file 2 — Table S2 Topic guide for interviews [file 12471_2022_1740_MOESM2_ESM.docx]

**Table S2** Topic guide for interviews

| Topic | Key Questions |
| --- | --- |
| Introduction | - Can you tell me about your work? What do you do at [organisation name]? - What is your role in the care of patients with chronic heart failure? /Development of the RTA/use of the RTA |
| Innovation | - What was your role in the development of the RTA? - What is your opinion of the development of the RTA in your region? - What is the added value of the RTA compared to the national transmural agreement? |
| User | - Has the RTA changed your work? If so, in what way? How do you view this? - What effects of using the RTA do you observe? - Has the RTA changed patient care? If so, in what way? What do patients think about this? What do you think about this? - In your estimation, how large is the proportion of your colleagues (in your region) that use the RTA? Which healthcare professionals more or less? - Have you experienced obstacles during the implementation of the RTA? How did you address these? |
| Organisation | - How is the use of the RTA promoted in your region? By whom? - How are new employees and other employees trained with regard to the RTA? - What are the financial consequences of adopting the RTA? |
| Socio-political context | - To what extent does the RTA/LTA fit within existing regulations (e.g. financial structures and legislation) - Which party could facilitate the implementation of the RTA? How? - Are there regular reflections or evaluations on the RTA? What are the findings during? |
| Conclusion | - Is there anything we haven't discussed about the RTA that you think might still be important? - Who do we really need to interview? |
