# Supplementary material for: Use of regional transmural agreements to support the right care in the right place for patients with chronic heart failure—a qualitative study
Source: Neth Heart J. 2022 Dec 12;31(3):109–16. doi: 10.1007/s12471-022-01740-5 (PMC9742644; doi:10.1007/s12471-022-01740-5)
Supplement: Supplementary file 3 — Table S3 Extensive description of themes [file 12471_2022_1740_MOESM3_ESM.docx]

**Table S3** Extensive description of themes

| **Education**  The importance of education for primary care (PC) was frequently emphasized by participants. It was considered essential for treatment of chronic heart failure (CHF) in PC and often the first action that regions started after the development of the Regional Transmural Agreements (RTA). While no exact number of participation rates were mentioned, participants from several regions indicated that approximately half of all PC providers in their region followed additional education on CHF. Most participants reported that CHF is perceived as complicated to treat and that knowledge and awareness of CHF varies among PC providers. Simultaneously, knowledge of the pathophysiology of CHF and awareness of CHF among PC providers were mentioned as important preconditions to deliver the care according to the NTA and guidelines. An important reason for a lack of awareness and experience was the number of CHF patients per PC clinic, which was often limited to approximately 10 to 15 patients. This limited number of CHF patients per PC clinic was also considered an important barrier for PC providers to follow additional education.  Education for PC providers was primarily aimed at refreshing and expanding knowledge concerning the pathophysiology of CHF and increasing awareness. In addition, many regions had secondary aims with educational sessions such as informing PC providers on care pathways, consultation abilities, and changes due to the RTA. Several cardiologists remarked that they actual had noticed the effect of education on the questions that PC providers asked and improved information collection and sharing. This resulted in more effective consultations by decreasing the number of diagnostic requests e.g. a consultation without reporting the brain natriuretic peptide levels. Most participants mentioned the importance of easy consultation options to mitigate knowledge and experience gaps.  We encountered a variety of ways in which PC providers had been educated. Examples were an evening class, e-learnings and on-the-job training but also spending a day with the nurse practitioner specialised in CHF in the hospital. Each of these examples had their own advantages and shortcomings. For example, an e-learning was an easy way for knowledge dissemination, yet did not facilitate personal relationships.  **Referral from SC to PC**  The National Transmural Agreement (NTA) provides guidance for the development of RTAs. However, the NTA does not impose certain criteria and gives regions the freedom to develop their own tailored agreements. This resulted in different RTAs and also in different criteria for referral back to PC. Whereas some regions strictly applied the criteria described in the NTA, other regions started with more stringent criteria but later loosened these once cardiologists and PC providers became more comfortable with referring patients with stable CHF back to PC. An example of this was a region which also referred patients with an implantable cardioverter defibrillator back to PC. While these patients were not suggested by the NTA to be referred back to PC, the hospital opted to partially transfer these patients to PC thereby reducing the number of times the patient visited the hospital (“shared care”).  Most participants mentioned that the criteria in the RTA were the most important determinants in the decision if the patient was referred back to PC. However, apart from criteria in the RTA, trust in the competencies within PC sometimes affected referrals. Several cardiologists noted that they were unaware of which PC clinic had affinity and experience with CHF patients and which PC clinic did not. In addition, several cardiologists reported that they had bad experiences with referring patients back to PC. This resulted sometimes in a lack of trust in PC skills which resulted in cardiologists not referring patients back to PC. However, most cardiologists reported that they followed the criteria in the RTA, regardless of the PC clinic. Most cardiologists and nurse practitioners tried to provide clear instructions for PC providers and, if possible, call with the PC provider before referring the patient back.  **Patients**  Several participants mentioned that patients are sometimes unwilling to be referred back to PC due to the high satisfaction with the care at specialised CHF clinics. They also remarked that this can be prevented by improving the education on the care pathways by both PC providers and cardiologists. By informing the patient in a timely and adequate manner the patient is prepared that he or she will eventually be referred back to PC. In addition, easily accessible consultation options of the CHF clinic for the PC providers and patients can improve the willingness of patients to be referred back to PC.  **Relationships**  Participants reported that RTAs must be in line with the skills and facilities of both PC and SC providers. These differed across but even within regions. For example, in one region one hospital provided a possibility to diagnose CHF for patients in PC, without an actual transfer of the patient to SC, while an other hospital in the same region did not provide this option. In some regions, some PC providers were able to interpret reports of echocardiograms themselves whereas this was more rare in other regions. These differences affected the way things were organised within the regions. Participants noted that it was important that developers of the RTA had good relationships with their colleagues and acknowledge their abilities and inabilities. This remained important during the implementation to ensure continued participation and the signalling of possible barriers and was not limited to PC providers. For example, cardiologists that had initiated the RTA also needed to monitor if cardiologists, with other specialties than CHF, also referred patients according to the RTA.  In addition, it was frequently mentioned that ‘knowing each other’, was an important facilitator for collaboration This created a better understanding of each other’s abilities and challenges and resulted in increased trust in each other. In addition, knowing each other increased willingness to refer and accept the transfer of patients from SC to PC. One way to facilitate ‘knowing each other’ was trough the education process. These personal contacts became scarcer as more healthcare providers were present within the region. However, once healthcare providers had participated previously in transmural projects they were more familiar with the organisations, processes and people involved which acted as positive reinforcement for future projects.  **Reimbursement**  Reimbursement of additional activities due to the RTA was experienced as a major hurdle by many participants. These additional activities referred to education, identification of patients, and (administrative) support for implementation. Whereas some regions achieved reimbursement for all additional activities, other regions did receive partial reimbursement or none at all. Healthcare providers reported that they were often unfamiliar with the processes regarding reimbursement and experienced them as complicated. Moreover, several healthcare insurance companies had different policies regarding if and how reimbursements were arranged. A lack of reimbursement for certain activities resulted in some participants refusing to participate in RTAs. A facilitating factor for receiving reimbursement was the presence of a well-organised care group and/or other umbrella organisation to support the administrative processes.  Problems with reimbursement often arose when business cases were developed. Participants reported that the shift of health care consumption, and thus also monetary streams, was a complex part of the business case. This not only required PC to provide additional healthcare but also SC to accept a decline in revenue. In addition, one participant reported that the number of patients that ultimately can be referred back to each PC clinic is probably limited whereas the additional costs are significant due to the large number of PC providers that need education and to scan their EHR for the identification of patients. However, several participants explicitly mentioned that healthcare insurers were too much focused on the monetary effect and should not forget additional benefits such as improved diagnosis and palliative processes and improved communication between PC and SC.  Besides a positive business case, warm relationships and trust between healthcare providers and healthcare insurers were mentioned several times as important factors by both healthcare providers and healthcare insurers. Several participants mentioned that they were insecure concerning future reimbursement.  **Electronic health record systems**  The ability to share data with professionals in other settings was perceived as a facilitating factor for the implementation of the RTA and improving quality of care. Where data sharing was not possible, participants reported that the lack of interoperability or different EHR systems as a complicating factor. This complicated information exchange between providers and hindered evaluations. Despite these interoperability difficulties, several regions had implemented additional consultation options through which patients could be diagnosed by a cardiologist without transferring patients to the hospital.  Identification of patients with CHF formed an important aspect of RTAs. This was not limited to the identification of stable patients within SC that may be referred to PC but also referred to undiagnosed patients within PC. Participants noted that time-consuming manual review of patient records was necessary to find a limited number of patients. In some cases, this identification process was supported by a care group or software solutions. For example, a software solution was used to identify certain high-risk patients based on administration codes.  **Outcomes**  Most regions performed evaluations on the effects of the RTA. However, the selection of outcome measures were heterogenous. The number of referrals to PC was often evaluated as part of reimbursement agreements and showed different results. Whereas some participants noted that initial targets had been achieved, others reported that initial expectations had been too high. Other outcome measures that were part of evaluations were process indicators such as the registration of physiological parameters. In addition, qualitative evaluations were used to identify areas for improvement. Several participants mentioned that data on provider level could be used to guide and motivate PC clinics by providing mirror information. While regions intended to evaluate patient experiences and satisfaction, little information was available. One region reported to struggle with the methodological, legal and practical obstacles to retrieve this information. A major hurdle for the collection end evaluation of certain outcomes was the lack of interoperability in EHR systems. |
| --- |
